# Management of antipsychotics in primary care: Insights from healthcare professionals and policy makers in the United Kingdom

**DOI:** 10.1371/journal.pone.0294974

**Published:** 2024-03-01

**Authors:** Alan A. Woodall, Aseel S. Abuzour, Samantha A. Wilson, Frances S. Mair, Iain Buchan, Sally B. Sheard, Paul Atkinson, Dan W. Joyce, Pyers Symon, Lauren E. Walker

**Affiliations:** 1 Department of Primary Care and Mental Health, Institute of Population Health, University of Liverpool, Liverpool, United Kingdom; 2 Powys Teaching Health Board, Bronllys Hospital, Powys, United Kingdom; 3 Unit for Ageing and Stroke Research, School of Medicine, University of Leeds, West Yorkshire, United Kingdom; 4 Department of Public Health, Policy and Systems, Institute of Population Health, University of Liverpool, Liverpool, United Kingdom; 5 General Practice & Primary Care, School of Health and Wellbeing, University of Glasgow, Glasgow, United Kingdom; 6 NIHR Mental Health Research for Innovation Centre, University of Liverpool, Liverpool, United Kingdom; 7 Department of Pharmacology and Therapeutics, Institute of Systems, Molecular and Integrative Biology, University of Liverpool, Liverpool, United Kingdom; University of Connecticut Health Center: UConn Health, UNITED STATES

## Abstract

**Introduction:**

Antipsychotic medication is increasingly prescribed to patients with serious mental illness. Patients with serious mental illness often have cardiovascular and metabolic comorbidities, and antipsychotics independently increase the risk of cardiometabolic disease. Despite this, many patients prescribed antipsychotics are discharged to primary care without planned psychiatric review. We explore perceptions of healthcare professionals and managers/directors of policy regarding reasons for increasing prevalence and management of antipsychotics in primary care.

**Methods:**

Qualitative study using semi-structured interviews with 11 general practitioners (GPs), 8 psychiatrists, and 11 managers/directors of policy in the United Kingdom. Data was analysed using thematic analysis.

**Results:**

Respondents reported competency gaps that impaired ability to manage patients prescribed antipsychotic medications, arising from inadequate postgraduate training and professional development. GPs lacked confidence to manage antipsychotic medications alone; psychiatrists lacked skills to address cardiometabolic risks and did not perceive this as their role. Communication barriers, lack of integrated care records, limited psychology provision, lowered expectation towards patients with serious mental illness by professionals, and pressure to discharge from hospital resulted in patients in primary care becoming ‘trapped’ on antipsychotics, inhibiting opportunities to deprescribe. Organisational and contractual barriers between services exacerbate this risk, with socioeconomic deprivation and lack of access to non-pharmacological interventions driving overprescribing. Professionals voiced fears of censure if a catastrophic event occurred after stopping an antipsychotic. Facilitators to overcome these barriers were suggested.

**Conclusions:**

People prescribed antipsychotics experience a fragmented health system and suboptimal care. Several interventions could be taken to improve care for this population, but inadequate availability of non-pharmacological interventions and socioeconomic factors increasing mental distress need policy change to improve outcomes. The role of professionals’ fear of medicolegal or regulatory censure inhibiting antipsychotic deprescribing was a new finding in this study.

## Introduction

Psychiatrists usually prescribe antipsychotic medication (APM) to treat serious mental illness (SMI) such as schizophrenia and bipolar affective disorder. Despite treatment, patients with SMI die 10–20 years prematurely, mainly from cardiometabolic diseases and cancer [1–5]. In recent years, ‘atypical’ APMs are increasingly prescribed due to fewer extrapyramidal effects [6], but they can increase risk of obesity and cardiometabolic disease [1,7], and require physical health monitoring, including weight, blood pressure, lipid and glucose levels, and for abnormalities on electrocardiogram (ECG) [8–10]. While they are licensed for treating psychotic illness, APM prescribing is increasing for personality disorder [11], depression [12], anxiety [13], insomnia [14], dementia [15], learning disability [16] and autistic spectrum disorder [17]. Currently, in the UK, only quetiapine is licensed as a treatment for depression, and risperidone is licenced for behavioural management in patients with dementia; other uses are off-label [18]. In some countries (e.g., Norway), APM is often initiated by GPs [19]. In the UK, APM initiation is usually undertaken by psychiatrists; where the patient remains under secondary care (psychiatric clinic or hospital), psychiatrists optimise APM, and GPs manage cardiometabolic risks [20,21]. Unfortunately, many patients with SMI who attend psychiatric appointments are less likely to see GPs for monitoring [20,21]. Many health systems, mainly designed to manage acute illness, struggle to provide continuity of care to patients with SMI [22]. Patients taking APM deemed stable from a psychiatric perspective, or those without psychotic illnesses, are regularly discharged by psychiatrists to be managed by GPs, which potentially creates gaps in care [23]. APM prescribing is increasing [6]; 32% of patients with SMI do not have psychiatric review [24]. Furthermore, patterns of disease presentation in populations are changing. More patients present with multimorbidity (≥ 2 chronic illnesses) and polypharmacy (≥ 5 medications) [25] and require co-ordination between specialities, professionals, stakeholders, and services to deliver optimum benefit, which is defined as ‘integrated care’ [26]. Patients with co-morbid physical and mental illnesses have poorer outcomes when care is fragmented [27]. A Cochrane review suggests outcomes are improved when collaborative care exists for management of depression and anxiety [28]. Studies examining collaborative care between GPs and psychiatrists have shown promising results in Denmark [29], and in Norway [30]; where psychiatrists and psychologists were co-located in general practice to promote collaborative care, these demonstrated improvement in care for patients with mental illness, but were unsustainable due to costs and contractual difficulties. Integrated care is often conflated with ‘shared care’, but they are not synonymous. Shared care plans (SCP) in the UK are a formal agreement between primary and secondary care to transfer clinical activity to GPs (usually management of medications with specialist advice, most commonly in physical illnesses [31]); the patient remains under specialist care and cannot be discharged while taking specialist medications [32]. Some SCP exist between psychiatry and GPs (e.g., to prescribe medications for Attention Deficit Hyperactivity Disorder) but APMs are not included. This study explores the management of patients taking APM discharged to primary care. We explored the views of healthcare professionals (HCPs) who deliver this care and managers or directors of policy (MDPs) of organisations where APM prescribing occurs in the UK. The aim of this study was to identify barriers and facilitators of care for people taking APM and views on the causes of increasing APM prescribing.

## Methods

### Design and setting

This study explored views of professionals and policymakers involved in care of patients prescribed APM in primary care. We conducted semi-structured interviews with participants recruited from the UK, using interview methodologies outlined by Linton and Ryan [33] in *Qualitative Research In Health Care* [34]. The ‘Consolidated criteria for reporting Qualitative Research’ also guided study design [35].

### Participants and recruitment

Participants were recruited from England, Scotland, and Wales between 1^st^ July 2022 and 31^st^ March 2023. These included GPs and psychiatrists in clinic and hospital settings, including rural and urban locations, and areas serving affluent or deprived populations. Some clinicians also held roles as clinical or policy directors. In addition to HCPs, service managers, government advisors and policy directors from stakeholders (e.g., mental health charities, Royal Colleges overseeing postgraduate training) were invited. Purposive sampling identified potential participants, who were sent study information and an invite to participate. Up to three invites were sent, after which recruitment attempts ceased. Participants were briefed about the study by researchers and written consent obtained prior to interview. Participants could withdraw consent at any time.

### Data collection

Semi-structured interviews were undertaken between July 2022 and May 2023, and details of participant characteristics (professional role, postgraduate experience of psychiatry and general practice, and duration of professional registration) were obtained. Interview schedules were developed and refined by the team after pilot testing (S1 File). Interviews were conducted by AW (clinician with qualitative research experience) and explored views on management of APM, barriers to care and causes of increasing use. Interviews were conducted via Microsoft Teams. Audio data was transcribed verbatim and anonymised; each participant was assigned a code, then recordings destroyed. Transcripts were not provided to participants for checking and no withdrawal of consent occurred after interview. Data collection and analysis were undertaken concurrently.

### Data analysis

Transcripts were analysed using inductive thematic analysis (ITA) as outlined by Pope et al [36] employing Braun and Clarke’s ‘six steps’ methodology [37]. QSR NVivo® Windows (Version 1.6) was used to organise, code, and analyse transcripts. Transcripts were repeatedly re-read to familiarise researchers with the data and note down initial ideas at regular team meetings. This was followed by reviewing sentence and paragraph segments from the transcripts to generate initial codes which used respondents’ exact words. The codes were then categorised into potential subthemes to organise the substantial number of codes initially generated. Data collection and analysis occurred concurrently. This allowed the researchers to constantly recheck the initial codes and subthemes generated inductively, to identify patterns within the data. This included comparing data against the codes and subthemes generated to organise concepts that clustered together to form larger themes, and to ensure the final themes generated reflected meaning within the data. The iterative categorisation of data into themes and subthemes, and interpretation of the data were undertaken by the coding team (AW, AA, SW, LW) to ensure analytical rigour. Data saturation was assessed via analysis of codes and themes, rather than at the point of data collection. Saturation was considered achieved when no new codes or meaning were being identified from the data [33,36], guided by assessment of saturation outlined by Saunders et al for ITA [38]. A systematic review of empirical studies show saturation is reached with 9–17 interviews [39]; for three heterogenous studies larger sampling was needed, but even these achieved saturation with 16 interviews. Only using ‘codes’ to determine saturation risks missing ‘meaning’; Hennick demonstrated that ‘code’ and ‘meaning’ saturation was achieved in a study employing ITA with 16–24 participants [40]. These numbers provide a guide for saturation assessment, but our main criterion to determine saturation was unanimous agreement that no new codes or meaning were emerging [41].

### Patient and public involvement

A member of the public with an interest in mental health care provided layperson oversight of the study.

### Ethical approval and governance

The study received ethical approval from the Health Research Authority (IRAS: 311503) via NHS Research Ethics Committee 6 (22/WA/0173).

## Results

Participant characteristics are shown in Table 1. As some MDPs have unique national roles, certain characteristics are withheld to maintain anonymity. Of 46 subjects approached (20 GPs, 13 psychiatrists, 13 MDPs), 30 subjects (11 GPs, 8 psychiatrists, and 11 MDPs) undertook interviews, lasting 21–58 minutes. Twelve out of thirty participants were female. The time on the register shows how long the clinician has been qualified as a HCP, rather than as a psychiatrist or GP. Some participants had considerable experience in their speciality (e.g. GP-06, PSY-03), while others were relatively newly qualified (e.g. GP-07, PSY-07).

**Table 1:** Characteristics of participants.

Participant ID	Role	Sex	Postgraduate UK training experience in psychiatry and general practice?	Time on UK professional register
GP-01	GP partner	Male	Yes	21 years
GP-02	Salaried GP	Male	Yes	22 years
GP-03	GP partner	Female	Yes	16 years
GP-04	Salaried GP	Male	Yes	16 years
GP-05	GP partner	Male	Yes	21 years
GP-06	GP partner	Male	No	32 years
GP-07	Salaried GP	Male	Yes	8 years
GP-08	GP partner	Female	Yes	21 years
GP-09	GP partner	Male	No	11 years
GP-10	GP partner	Female	Yes	20 years
GP-11	GP partner	Female	Yes	21 years
PSY-01	Consultant Psychiatrist	Male	No	19 years
PSY-02	Consultant Psychiatrist	Male	No	25 years
PSY-03	Associate Specialist in Psychiatry	Male	No	30 years
PSY-04	Consultant Nurse in Psychiatry (Responsible Clinician)	Male	No	27 years
PSY-05	Consultant Psychiatrist	Male	No	21 years
PSY-06	Consultant Psychiatrist	Female	No	17 years
PSY-07	Specialty doctor in Psychiatry	Male	No	10 years
PSY-08	Consultant Psychiatrist	Male	No	22 years
MDP-01	Former educational advisor (psychiatry) to national postgraduate training committee and Royal College of Psychiatrists	Withheld	No (also a consultant psychiatrist)	Withheld
MDP-02	Policy director, mental health charity	Withheld	N/A	N/A
MDP-03	Government advisor on mental health	Withheld	Withheld	Withheld
MDP-04	Former executive officer of Royal College of General Practitioners	Withheld	Yes (also a GP)	Withheld
MDP-05	Medical Director of Local Medical Committee for health region	Male	Yes (also a GP)	Withheld
MDP-06	Director of Mental Health Services for provider organisation	Withheld	Withheld	Withheld
MDP-07	Director of Primary Care for a provider organisation	Withheld	Withheld	Withheld
MDP-08	Chief Pharmacist for a provider organisation	Withheld	Withheld	Withheld
MDP-09	Medical Director for a provider organisation	Male	Yes (also a GP)	Withheld
MDP-10	Representative of General Practitioners Committee (British Medical Association)	Withheld	Yes (also a GP)	Withheld
MDP-11	Former executive officer of General Practitioner Committee of British Medical Association	Withheld	Yes (also a GP)	Withheld

Five themes emerged that affected APM management in primary care: Theme 1—Confidence of HCPs around management of APM; Theme 2—Service pressures ‘trapping’ patients on APM in primary care; Theme 3—Communication barriers between primary and secondary care; Theme 4—Lowered expectations of patients by HCPs; Theme 5—Strategic factors, including contractual divisions between primary and secondary care, socioeconomic inequality causing mental distress, and medicolegal fears of HCPs, affected whether patients remain on APM. Themes (with illustrative quotes) are discussed, and additional supporting quotes are available (S2 File). Potential facilitators of care suggested by respondents are also discussed.

### Theme 1: Confidence of HCPs around management of APM

Care of patients taking APMs requires HCPs trained to manage both mental and physical health concerns. Three subthemes emerged:

#### Subtheme 1a: GPs were reluctant to manage APM without psychiatrist support

Most GPs reported their training did not encompass APM management and stated they lacked skills to manage APM without psychiatrist support. GPs were also concerned that patients lacked confidence in their ability to advise on APMs appropriately:

*“I don’t think that my [junior doctor psychiatric] training necessarily made me more confident in relation to antipsychotics*… . *I don’t think I’m confident enough to do that without some input*.*”* (GP-07)

*“It’s not just a GP thing*…*the patient might not have the confidence in us to do it either*… *They’ve often demanded to be referred to psychiatry*, *because the GP couldn’t make them better*… *to be then told by the same GP ’well actually*, *I’m going to stop your antipsychotic’- the patient may not have the confidence that stopping it will be OK*.*”* (GP-11)

Psychiatrists recognised GPs’ reluctance to manage APM when patients were discharged, but some psychiatrists stated that GPs should manage APM independently:

*“Your average GP has excellent capabilities to manage someone on antipsychotics*… . *The thing I think they lack*… *would be the confidence to do so*.*”* (PSY-01)

Potential facilitators to help GPs manage patients prescribed APM independently included support from psychiatric pharmacists optimise APM prescribing:

“Psychiatry pharmacists are very easy to contact… . they tend to give detailed letters as to…what you could do. There are alternatives. I wouldn’t necessarily make a decision straight away without just sending the pharmacists a quick letter to say, “this is a situation what would your advice be” and they might say “aripiprazole… .and then you need to do x, y, z”…. I don’t think I would just switch [APMs] myself because I don’t necessarily have the confidence or the knowledge to do that.” (GP-07)

#### Subtheme 1b: Psychiatrists were reluctant to address cardiometabolic risks

Respondents said psychiatrists often disregarded cardiometabolic risks. Psychiatrists explained they lacked the skills to address cardiometabolic risks, but there was also a reluctance to consider physical health as their responsibility:

*“I would feel unqualified to do that [manage a diabetic patient on their ward]*. *I don’t feel I’ve got the knowledge to be able to do that in a helpful way…*.*I would be pretty likely to be doing the wrong thing and causing more trouble*.*”* (PSY-03)

Respondents suggested this limited opportunities to ameliorate cardiometabolic risk for these patients. Psychiatrists acknowledged patients with SMI might not see their GP on discharge, and this put them at greater risk of poorer cardiovascular outcomes:

*“I received no training in general practice*… *that’s never been part of my psychiatric training*. *The idea that I would comfortably manage hypertension*… *I don’t feel comfortable with it”* (PSY-07)

*“It’s all ’go and talk to your GP’…and the patient often doesn’t go*.*”* (MDP-10)

GPs understood psychiatrists’ reluctance to initiate medications for cardiometabolic risks but criticised a lack of non-pharmacological interventions (e.g., referrals to dieticians), and expected GPs would undertake these. GPs also noted that psychiatrists rarely made APM switches to ameliorate cardiometabolic risks, their focus being on addressing psychiatric issues:

*“There is*…*mention in some letters about us helping the patient control lifestyle factors*… *What I don’t [see] is any evidence in letters that those referrals have been made by the psychiatric teams themselves*.*”* (GP-11*)*

*“Your focus tends to be on mental health*…*when you’re seeing someone in their 20s [with] acute psychotic illness and their life is coming apart—you’re focusing on that*, *and you are thinking ‘they might die of heart disease when they’re 60*? *If I get them to 60*, *I’ve done really well*!*’ “*(PSY-01).

*“I think the GP can recommend or prescribe exercise as well*…*I’ve never done it*…*when I came here*, *I was [told] ’you ask the GP to do that’*.*”* (PSY-08)

Respondents suggested integrating GPs into mental health teams, particularly for inpatient settings, would overcome psychiatrists’ reticence regarding physical health, and ensure that inpatients had physical health needs met before discharge. Others proposed that support workers to co-ordinate physical health monitoring would help in community psychiatric teams:

*“Either we make primary care accessible to that group of people or we embed generalists within mental health… it’s going to be probably easier to embed people with good general physical health*… *intervention skills within mental health*, *because we know that our group of patients… wouldn’t necessarily present to a GP”*. (MDP-06)

#### Subtheme 1c: Gaps for HCPs in postgraduate and post-speciality training

HCPs reported inadequate training to manage both psychiatric and cardiometabolic aspects of APMs. While all but two GPs interviewed had undertaken postgraduate psychiatric training, no psychiatrist interviewed had postgraduate experience in general practice (Table 1). This impaired understanding of each other’s specialties: some psychiatrists thought that all GPs undertook psychiatric postgraduate placements, and this drove expectations they could manage APMs. Psychiatrists confirmed postgraduate GP training did not cover APM management:

*“I think [GP trainees] were*… *treated differently [to psychiatric trainees]*. *It was felt that they didn’t need that sort of in-depth knowledge [around APM management]*.*”* (PSY-05)

*“GPs would have all had to have done some training in [psychiatry]… am I wrong in that*? *[informed <50% of GPs have psychiatric placements] … I’m actually shocked…*. *I had the impression that they [all] had to do*… *psychiatry*.*”* (PSY-04)

GPs were concerned that stakeholders wanted them to manage more patients on APM without appreciating the volume of specialist care being transferred to GPs:

*“Special interest groups are saying*, *’Well*, *actually*, *this is not complicated*… *if you do some online teaching for an hour then the GP should know enough’…*. *It’s not possible*, *because we can’t keep up to date with all the different antipsychotics*… *as well as keeping up to date with all the different specialities and their specialist medications*.*”* (GP-11)

Psychiatrists reported they had little training on cardiometabolic risks of APM, and MDPs involved in training confirmed this gap in the psychiatric curriculum. One MDP (former director of postgraduate psychiatric training) suggested that psychiatric trainees should have opportunities to work alongside GPs to improve their physical health skills and learn how primary care functions:

*“It doesn’t seem to be built into the curriculum*. *I don’t directly influence what’s going on in the [Royal College of Psychiatrists] anymore*… *I don’t know what it says in terms of speaking to physical health*… . *for adult psychiatry trainees*… . *[we need] to give them more experience of working in*… . *primary care*.*”* (MDP-01)

There was a concern by GPs and psychiatrists that psychiatric training placements for GPs were often unsuitable to gain relevant experience (e.g., GP trainees placed in forensic hospitals) and both groups said there was little recognition by their Royal Colleges (who oversee postgraduate training) that management of APM was increasingly transferred to GPs. They suggested this should be addressed by curriculum co-ordination between the Royal College of General Practitioners (RCGP) and Royal College of Psychiatrists (RCPsych). Others suggested changes within the RCPsych were needed to accept that physical health concerns arising from APMs was their responsibility::

*If it’s a sequelae from their medications*, *I think they should be training on looking at ECGs and looking for QTc abnormalities*, *management of diabetes and [cardiovascular risk assessment]*… . (MDP-05)

GPs reported training sessions with psychiatrists were needed, to look at integrated care of patients on APMs, reflecting the need for both specialities to develop joint strategies to improve integrated care:

*“I would love to see our [training] sessions return to…group-based multidisciplinary sessions*… *It’s a brilliant opportunity that we’ve got to develop beyond primary care*.*”* (MDP-09]

### Theme 2: Service pressures ‘trapping’ patients on antipsychotics in primary care

Respondents postulated service pressures had increased APM prescribing, with three main subthemes emerging:

#### Subtheme 2a: Limited psychology provision

Respondents expressed that limited psychology provision drove APM prescribing as a ‘stop-gap’ which was not good practice:

*“What do you do when someone is presenting as severely anxious*, *mood difficulties*, *not sleeping*, *incredibly chaotic*: *wait 18 months for therapy*?… *Do you simply adhere to NICE recommendations and say*… *‘there’s nothing I can do for you*, *you’re going to have to wait 18 months*, *it doesn’t matter how distressed you are*?*”* (PSY-08)

One respondent suggested assistant psychologists could help manage psychological need, as so few fully trained clinical psychologists were available:

*“Some of these newer roles—the Clinical Associate in Applied Psychology*… *they can work with people in the psychological model under supervision*. *They can do the psychology*, *the formulations*, *they can deliver the interventions with support*…*”* (MDP-01)

#### Subtheme 2b: Pressure to discharge patients to primary care

Psychiatrists reported pressure from management to discharge patients on APM, including those with SMI if stable, and especially patients without psychosis. As a result of this transfer of care, GPs were concerned patients would be unlikely to ever stop taking APM:

*“There was a lot of pressure a few years ago on caseload cleansing…*. *the only ones we [were told to keep] were the ‘complex and unstable’…*. *this is ridiculous*… *if I discharged [the other patients] to primary care*, *they’d be back in within two weeks*.” (PSY-01)

Many psychiatrists were also uncomfortable with discharging these patients, and suggested patients with SMI should remain under their care to have annual reviews. Psychiatrists and GPs often found it difficult to manage cardiometabolic risk, due to lack of resources to undertake monitoring and interventions–a lack of time, clinic space, or clinical equipment were reported as barriers:

*‘Where I work*, *we don’t have any ECG machine*, *so I’m not able to just get on and do it myself*, *or train up the staff*…*’* (PSY-02)

Respondents suggested many potential facilitators to reduce pressure to discharge patients to primary care. Managers proposed removal of numerical targets imposed by governments as a measure of efficiency–the preference being to measure quality of outcomes. GPs wanted SCPs for antipsychotic prescribing, with discharge from psychiatry only undertaken with mutual agreement:

*“Secondary care often seems to think they can then just discharge patients [on APM] without any agreement*… *the problem is that [they] don’t tend to attach any funding to it… I think the main difficulty is getting proper [SCPs] in place*, *with the funding*… *and [then] you’ve then got the resources to do it properly*.*”* (MDP-05)

#### Subtheme 2c: Difficulty to get psychiatric review for patients with cardiometabolic symptoms

GPs reported difficulty obtaining psychiatric review when cardiometabolic risks developed in patients taking APM discharged to primary care. Psychiatrists often advised ‘no APM change’ due to pressure on appointments rather than see them again, so opportunities to address cardiometabolic risk (e.g., switching APM to ameliorate risk of weight gain) were missed due to resource limitations:

*“There’s time pressure -if I’ve got someone [psychiatrically] stable and want to switch their antipsychotic*, *then I’ll have to give them more clinic appointments*.*”* (PSY-02)

As an alternative to formal SCPs, some respondents suggested 5-yearly comprehensive reviews of patients taking APM in primary care. There was some difference of opinion amongst respondents as to whom would be best placed to undertake reviews. Suggestions including training GPs with an interest in mental health, psychiatric nurses, or pharmacists. Psychiatrists however, suggested that it was unlikely other professionals would have the confidence to withdraw APM, so they should undertake them:

*“It would need to be done by the person with the most well-developed sense of judgment of risk*… . *[there is] a strong case to argue for it being a psychiatrist doing it*… . *You could get somebody else doing it*, *but if they were less confident*, *taking somebody off [antipsychotic] medication is always a bit of a scary thing to do*. *And it is always kind of tempting not to*, *because*…*you know what risks there are*…*”* (PSY-03)

### Theme 3—Communication barriers between primary and secondary care

Respondents reported that inadequate communication, both written, verbal, and in terms of Information Technology (IT) functionality, as three subthemes which impeded care of patients prescribed APMs:

#### Subtheme 3a: Inadequate information in written handover

HCPs reported inadequate information in psychiatrist’s letters on APM indication, duration, and monitoring; GPs were often unaware when APMs were prescribed ‘off-label’. GPs reported written advice was usually when to increase APM doses to avoid referral back to psychiatry. This left GPs with little confidence to optimise APM prescribing:

*“As someone who completes discharge summaries*, *I’m going to hold my hand up and say*, *we don’t always include that… GPs get patients discharged on an antipsychotic when they’re 23 and they’re still on the same antipsychotic at 43” (*PSY-01)

*“One case that I saw very recently*, *which was an elderly gentleman*… . *his psychiatry team said he’s stable enough to reduce his [APM] and discharge*… *but didn’t then give any sort of plan to reduce it*.” (GP-07)

Psychiatrists reported referral letters from GPs lacked detail; lack of personal knowledge of the patient in referrals inhibited care, often only being a computer-generated summary:

*“Referral quality can be variable depending on the experience of the GP*, *and their interest in mental health*… *sometimes we [have to] go back and ask for more information*.*”* (PSY-05)

Most respondents suggested psychiatrists having standardised letter templates that provided clear information on indication, intended duration, and plans for switching, weaning and reduction, would help support GPs to manage APM. Many noted that the only advisee was on dose titration, and this was insufficient:

*“It would be useful if the [psychiatrists] said they’re on this drug*, *which requires [this] monitoring*, *and we would expect them to stay on the drug for [this] length of time*. *And I think they should say what the indication is and that it’s being used off-[label]*.*”* (GP-10)

#### Subtheme 3b: Difficulties in verbal communication between primary and secondary care

GPs reported difficulties getting to speak to psychiatrists, with responses often from other professionals unable to advise on APM use; psychiatrists were concerned GPs did not often know patients well enough to provide any useful context when they called to find out more about the patient:

*“You often find—no disrespect -… queries that we raise are dealt with by health care workers way below the level of training of a consultant psychiatrist*.*”* (GP-11)

*“Because of the nature of general practice*, *you [often cannot] talk to a GP that knows the patient personally*… *information that comes with the familiarity*… *can be difficult to access*.*”* (PSY-07)

Some respondents suggested services such as ‘Consultant Connect’ [42] were helpful, where a GP could speak to a psychiatrist for advice, but because they could be located anywhere in the UK and did not have access to the EHR, their value was limited regarding advice on APM. Others suggested that a ‘consultant of the day’ rota providing advice to GPs would improve care, as they would have EHR access to guide management more effectively. Psychiatrists also wanted direct telephone access to GPs to improve communication. An indirect communication benefit from joint training was also proposed—teams would develop closer links to improve mutual trust, problem-solving, and communication:

*“We developed a ’psychiatrist of the day’ who would simply take calls from GP’s*, *and it was amazing*: *the upskilling that results from that*. *The value of a local connection is twofold*: *One is*, *as a GP*, *I can seek advice about altering some of these medications*, *or whether or not they need referral*?… *Two*, *also the ’psychiatrist of the day’ gets to pick up what are the issues going on out there… ‘Do we need an education event*, *or a change to a pathway*?*’”* (MDP-09)

#### Subtheme 3c: Poor integration of IT systems used in health services

Respondents stated that suboptimal IT systems inhibited ability to manage patients taking APM, due to different EHR systems being commissioned by primary and secondary care. HCPs often reported inability to view EHRs, wasting time when seeking information:

*“The hospital will have one electronic system*, *and the GP will have a second electronic system*. *And then psychiatry has a third*, *even more separate*, *difficult to access system*, *which some people have viewable access to*, *but not editable access*.*”* (GP-07)

All respondents wanted improved access and ability to write into a common EHR to avoid duplication of effort and aid integrated care planning. Respondents recognised with widening access that patient confidentiality was a concern, but some suggested that an overemphasis on patient confidentiality was blocking vital access, and having multiple EHR systems within services, led to fragmentation of care:

*“That kind of holy grail of the shared care record*, *it would sort it all out*, *it would solve it all*.*”* (GP-08)

### Theme 4: HCPs have low expectations of patients taking APM

Some HCPs expressed low expectations of patients with SMI. They found some difficult to engage with and complained patients often missed appointments, causing frustrations. One suggested this was due to lack of motivation in these patients to take responsibility for their health. Other HCPs, however, postulated that these patients were disadvantaged by the health system, often faced discrimination, and were given insufficient information on APM to enable informed consent. This encouraged them to advocate for their patients:

*“They are not people who ask for help*…*they’re beaten down by the system as well*, and they accept their lot is just s***, *and ‘that’s just the norm for me’*… . *“They are seen to be a lost cause…*. *‘Oh*, *they will never give up smoking*, *so there’s no point in me asking’*…. *And not only that*, *but it’s also their own fault—it’s the opposite of the truth*, *isn’t it really…They respond well to having someone who is interested in them*… *I’m not sure I make a massive difference*… *we’re there to pick stuff up when things go wrong*, *and start metformin when they inevitably become diabetic*, *or write the death certificate when they die young*. *You know*, *it feels a bit depressing to just verbalize it*.*” (*GP-08)

Many HCPs reported that addressing lowered expectations towards patients with SMI was challenging, and this mirrored societal attitudes. A director of a mental health charity suggested that more exposure of GPs to psychiatric training would help improve attitudes.

### Theme 5: Strategic factors affecting APM prescribing in primary care

Participants identified multiple professional and organisational constraints acting as barriers to care for patients taking APM. Three main subthemes emerged:

#### Subtheme 5a: Contractual divisions between primary and secondary care

Respondents expressed concern that the UK General Medical Services (GMS) GP contract inhibited integrated care:

*“The GMS contractual issues… that constant argument around "I’m not going to do this unless [we GPs] get paid for it"*. *Those sorts of issues really need to be addressed*… *sometimes [GPs] forget there’s a patient at the end of all these decisions*.*”* (MDP-08)

The Quality and Outcomes Framework (QOF) in the GMS contract incentivises GPs to monitor physical health risks for patients with SMI, but incentives have been largely abolished in contract revisions in some regions; GPs predicted this would reduce cardiometabolic risk monitoring without earmarked funding. GPs are not expected to provide specialist psychiatric care in their contract and complained services discharging patients with SMI or on APM to them to manage was an unfunded transfer of care. Many were concerned the QOF ‘mental health register’ criteria that drives monitoring, being focussed exclusively on psychotic illnesses, no longer captured all patients prescribed APM and was not fit for purpose, with a risk many patients were not recalled for monitoring. GPs suggested that APM use, not diagnosis, should trigger QOF funding for risk monitoring and payment in their QOF contract, rather than being limited to patients with psychotic illnesses:

*“In England*, *we still have the [QOF] mental health register…*. *but it doesn’t pick up people who’ve got a personality disorder [taking APM]*.*”* (GP-04)

Many respondents suggested a need for more collaborative working between Royal Colleges, medical unions, and service providers to identify barriers to integrated care, and amend contracts to overcome these, but felt organisational protectionism impaired progress. Primary and secondary care often worked in ‘organisational silos’ to create barriers to protect resources, e.g., referral to obesity clinics being restricted to GPs, and service managers needed to redesign and monitor pathways to prevent silos forming. A former RCGP leader reported ‘protectionist’ conflicts of interest even at national levels, where patient needs were secondary to the needs of the organisations competing for resources:

*“There is a real tension between being a member’s organization that needs to deliver for its members’ interests*, *and doing the right thing… I was there representing [the RCGP]*…*we can see what the right thing is to do*, *but as membership organizations*, *we have to protect the needs of our members*. *So*, *you would not expect the [RCGP] to meet with the [RCPsych] and [say] ‘You have all the money we get for mental health…just sort out everything’…protectionism gets in the way of better care*.*”* (MDP-04)

Some respondent*s* suggested patient pressure groups skewed resource allocation:

*“When*… *you’re taking a massive stack of antipsychotics*, *with all the negative impact that has on motivation*… . *those patients are the ones that I feel are most neglected by mental health services*… *the patient groups that talk the loudest*, *are [those] who we perhaps have the least value to offer*… .*”* (PSY-07)

MDPs expressed frustration that their efforts to reduce APM overprescribing did not result in savings to reinvest in services such as psychology:

*“Prescribing budgets are held in a different part of the [NHS organisation] … I can’t get hold of prescribing budgets*, *even if I’ve saved on one element of them*, *to reinvest the savings*.*”* (MDP-06)

#### Subtheme 5b: Worsening inequality driving APM use

Respondents suggested APM use was a ‘sticking plaster’ to deal with consequences of socioeconomic inequality and the medicalisation of distress, and many patients primarily needed social interventions regarding finances, housing, or education rather than health treatment:

*“Until we’re able to deal with the fact [people] can’t feed their kids*, *or heat their homes*, *that schools don’t have the resources to put into troubled young people*. *[antipsychotic prescribing] is not going to reduce in our medicalized society*… *I can absolutely see why [antipsychotic prescribing] rates are rising*.*”* (MDP-04)

Many respondents expressed a sense of powerless to address the socioeconomic determinants driving mental distress in patients, which could only be addressed by governments.

Another area of concern was pressure to prescribe APMs to manage challenging behaviours in people with dementia. Respondents said this was due to care home staff lacking the skills to manage patients with behavioural difficulties via non-pharmacological methods. They suggested investment in better training for care home staff would help to reduce APM prescribing:

*“Care homes are running on empty*. *They don’t have people who are skilled…*. *they have token specialist dementia champions*, *but they just don’t have the resources to sit with people while their anxiety levels reduce or engage them… [with the] things that we know…reduce the need for antipsychotic use*.*”* (GP-04)

#### Subtheme 5c: Medicolegal and regulatory fears of HCPs

HCPs mentioned fears around stopping APMs if a patient subsequently harmed themselves or others; some explained they ‘prescribe to be seen to do something’ due to working in an unforgiving medicolegal, regulatory, and societal culture:

*“If I was to stop an antipsychotic*…*if something adverse happened… I would have a very difficult time justifying that action*, *even if it’s a relatively safe thing to [do]*… *those things lead to medication overprescribing*… *even if it’s the wrong thing to do*, *it’s almost a sense that having done something is better than doing nothing*.*”* (GP-04)

Psychiatrists reported unrealistic societal expectations towards their speciality that an adverse outcome must ‘always be someone’s fault’, which inhibited their willingness to deprescribe:

*“Physicians accept that there’s a certain percentage of COPD patients who will die*, *that it doesn’t matter [how] much resource you use*…*they will get worse*, *and some of those people will die*. *And then there is a very marked reluctance… [within society] to accept that [psychiatric] conditions also have a morbidity and mortality*… *which I find frustrating*.*”* (PSY-07)

Many respondents suggested excessive regulation created barriers and encouraged defensive medicine. They mentioned that society, the judiciary and regulators had not recognised this, and wanted a debate around the increasingly adversarial ‘blame culture’ in which they had to practice.

One unintended consequence of overzealous national performance management indicators contributing to increased APM prescribing was close monitoring of benzodiazepine use, often reported down the level of identifiable clinicians, and some reported unease in case this resulted in censure. Some practitioners admitted ’gaming the system’ by using sedating APMs to treat insomnia to reduce their benzodiazepine rates, as, paradoxically this was not monitored at all:

*“I’m guessing if we use diazepam as an example—diazepam was the elixir for everything*. *And we had*, *going back*, *a massive uptake in the prescription of diazepam*. *We are aware of the issue of diazepam now has*, *in a sense of its addiction*… *so we just turn to other medication with a similar effect; in this case it is antipsychotics*.*”* (PSY-04)

## Discussion

**Fig 1:**
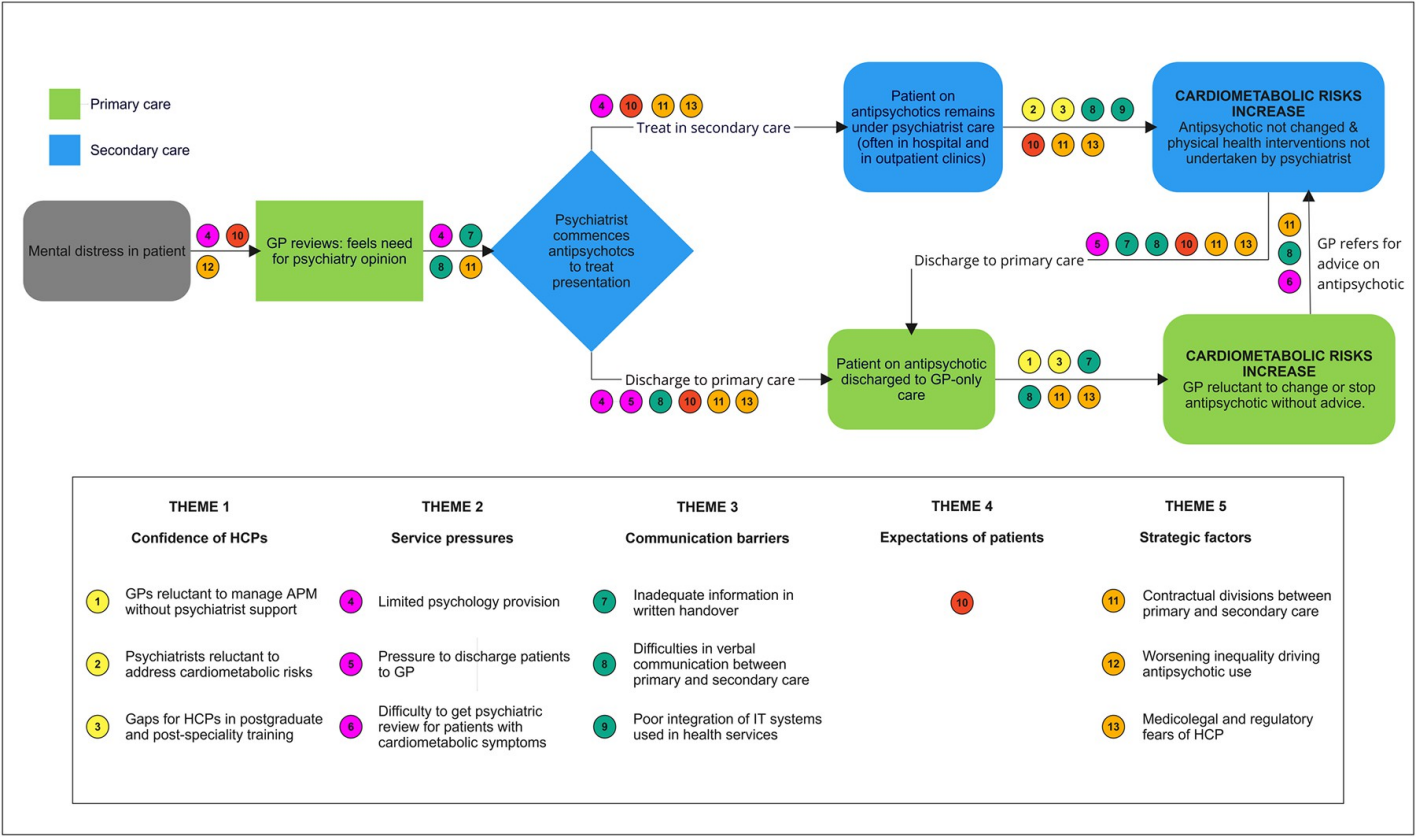
Pathway of patient care on antipsychotics and barriers to better integrated care.

This research shows how HCPs and MDPs reported competency gaps that impaired HCP ability to manage the concurrent mental and physical health needs of patients prescribed APMs. Stakeholders suggested that deficiencies in postgraduate experience contributed to GPs and psychiatrists’ lack of confidence to manage both aspects of APM management. Communication barriers, lack of common EHRs, limited resources, particularly in relation to psychology provision, worsening inequality leading to medicalisation of distress, and pressures to discharge patients to GPs all ultimately resulted in patients in primary care becoming ‘trapped’ on APM more often, limiting opportunities to deprescribe, and increasing their cardiometabolic risk in the longer term. Organisational, professional, and contractual conflicts between services working in ‘silos’ impeded collaborative care. Both GPs and psychiatrists voiced professional fears of being blamed if a catastrophic event causing harm occurred after stopping an APM, which impaired deprescribing. Some respondents had low expectations of patients with SMI, which may lead to less effort to help them improve their health. These factors act at several points in the care pathway and contribute to the increasing prevalence of APM use and accretion of cardiometabolic risk in this patient group in primary care (Fig 1).

This study has strengths and limitations. We had input from team members from different professions (medicine, pharmacy, health research), medical specialities (general practice, psychiatry, clinical pharmacology, public health) and the study sought views of those managing services, helping shape health policy and contractual arrangements, and overseeing postgraduate training. We recruited participants from three UK nations, which included urban, rural, affluent, and deprived areas, to try to minimise bias. 40% of participants were women, which is a reasonable reflection of those delivering healthcare, and we had a range of experience levels, from clinicians practicing in their speciality for a short period (<5 years) to those with considerable experience (>25 years). We believe data saturation was achieved as described, but in a heterogenous sample caution is needed, and there is the possibility other themes were not detected. Voluntary participation means respondents likely had an interest in mental health, and we may not have acquired views of other clinicians, so there is a risk of recruitment bias. Finally, this is a qualitative study that identifies perceived issues of concern in relation to APM management, but it does not provide information about the relative importance of the themes identified. Also, this work reports UK experiences; while there may be similarities with other countries, there may be differences worthy of exploration to better understand international issues. Finally, this study does not seek the views of patients who are prescribed APM in primary care (this is under investigation).

### Implications for clinical practice and research

**Fig 2:**
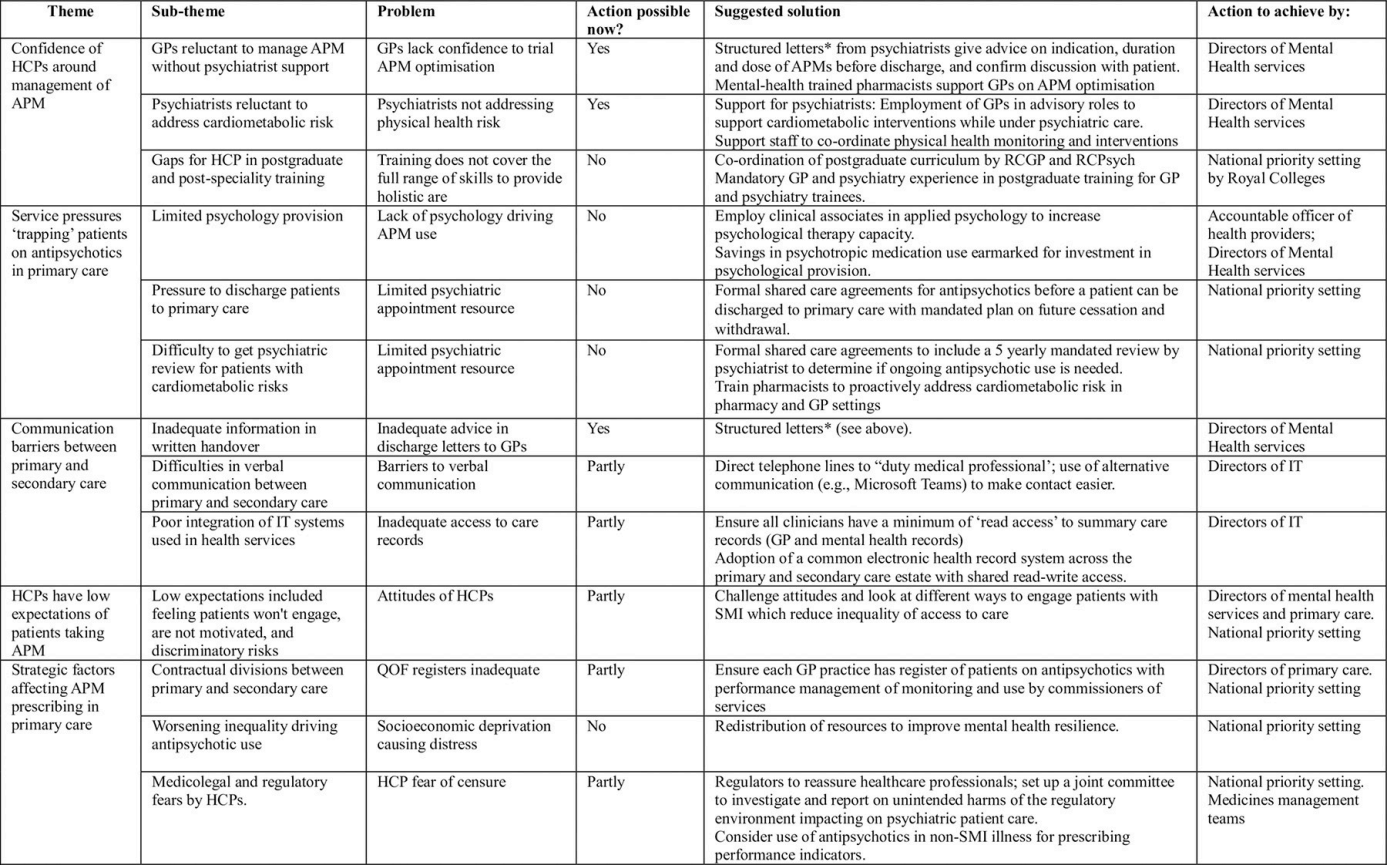
Potential actions to improve care for patients taking antipsychotics suggested by respondents.

This is the first study in the UK to directly examine stakeholder perceptions of management of APM for patients discharged to primary care and the reasons for increasing APM use, which makes comparisons difficult, but other studies partially consider this area. The study findings suggest several actions policymakers and practitioners can take now to improve person-centred care for people on APM (Fig 2), which we discuss across the themes and in comparison with published research:

### Closing the gaps in integrated care

Our analysis suggests that addressing competency gaps (theme 1) and communication barriers (theme 3) between primary and secondary care are those which are most amenable to change locally, but the causation of these care gaps are multifactorial (Fig 1). Grunwald et al [43] undertook a realist literature review to investigate how effective GP medication reviews are for patients prescribed APM. They found no studies that directly explored the needs of GPs or patients discharged to primary care, but identified several themes likely to act as barriers that we confirm, namely lack of confidence in GPs to reduce APM without supervision (theme 1), service pressures (theme 2), low expectations towards patients that they can stop APM (theme 4) and fears around risk of stopping the medication and subsequent harm to others (theme 5). Jakobs et al [44], while examining Dutch GP management of cardiovascular risk factors of patients with SMI or taking APM found similar themes: disproportionate burdens on GPs in deprived areas (themes 2 and 5), poor communication between GPs and psychiatrists (theme 3), scepticism amongst GPs that patients would comply with treatment, and ambivalence to apply risk-lowering interventions (theme 4). Nash et al [20] examined decision-making around APM switching by psychiatrists and GPs in one region in England but did not specifically consider patients managed solely in primary care. Their work and other studies conducted with patients and professionals in the Netherlands reported similar themes: concerns around jeopardising mental health by switching APMs to reduce cardiometabolic risk was mentioned as a reason not to do so (theme 5), poor engagement with patients to make informed decisions (theme 4), and diffusion of responsibility with respect to managing cardiometabolic risks secondary to APM use (themes 1, 2) [1,20,44]. Our data confirms these findings, and extends them, showing that issues including HCP training, contractual, organisational, medicolegal, and regulatory factors also function as barriers to integrated care. Further research is required to explore experiences of patients prescribed APMs discharged to primary care, and to examine if similar barriers to care exist internationally where primary and secondary care operate in an analogous manner to the UK (e.g. Ireland, Netherlands, New Zealand, Norway).

### Improving postgraduate training for GPs and psychiatrists

Review is needed of postgraduate education policy in the UK that is contributing to fragmented care. Psychiatry training is overseen by the RCPsych, involving a minimum of 3 years of ‘core psychiatric training’, and 3 years ‘higher speciality training’ focussed exclusively on acquiring one of six specific specialties [45]. The psychiatric curriculum has minimal physical health training exposure after foundation (intern) training all UK medical graduates undertake, and it is clear many psychiatrists worry they quickly lose physical health competencies. GP training takes 3 years and is overseen by the RCGP [46]. Trainees will, at most, only spend 4 months in a formal psychiatric placement, and over half of GP trainees have no psychiatric placement [47]. Both Royal Colleges need to ensure trainees receive comprehensive training to acquire skills to meet the future physical and mental health needs of patients who are prescribed APM. This is important as rates of multimorbidity in the population increase and fewer patients present with single illnesses. Most research has focussed on GPs being trained to manage multimorbidity better [48,49], but we suggest more HCPs need to acquire these skills (theme 1); psychiatric inpatients have considerable physical health data collated that allows accurate quantification of cardiometabolic risk, but these are not routinely acted on by psychiatric teams [50]; training to ensure cardiometabolic risk interventions are undertaken is needed for all psychiatrists (theme 1).

### Improving integrated care and communication between services to prevent patients becoming trapped on antipsychotics in primary care

Several studies in the UK, Netherlands and Canada confirm difficulties in communication (theme 3) and contractual barriers between primary and secondary care (theme 5) inhibit integrated care for people taking APM [20,51–54]. Implementing structured discharge letters (theme 3) that specify dose, duration, and eventual aim for deprescribing should be a priority for all services. SCPs, where the patient taking APM remains under the care of a psychiatrist, but overall day-to-day responsibility for prescribing and monitoring of the patient is contracted to the GP, would improve care. They are recommended in other medical specialties to improve care and reduce risk of medicines-related harm [32]. Morgan et al have suggested the need for mandatory SCA to achieve better health for those living with SMI [55]. We support this proposal and suggest a SCP for antipsychotics is considered which is of low burden to psychiatrists, where stable patients are mainly managed in primary care, but remain on a ‘virtual’ psychiatrist list that guarantees a mandatory 5-yearly review, and would hopefully prevent patients remaining on APM inappropriately in primary care. This would also overcome gaps in care that are emerging as the clinical use of APM expands beyond psychotic illness, and more patients treated with APM are missed from contractual QOF monitoring (theme 5). UK and international guidelines exist for the biological treatment of schizophrenia in primary care [56,57]. They partially guide the management of APMs in UK practice but require expanding to advise specifically on APM use in non-psychotic illness. There is an urgent need to reduce the use of APM for non-psychotic illness, where evidence for benefit is often limited. Care homes need staff trained to manage behavioural consequences of dementia without recourse to APM (theme 5); evidence suggests psychosocial interventions can reduce APM use in care homes [58,59]. Worsening socioeconomic inequality is strongly correlated with prevalence of mental distress and illness [60]. That can only be addressed by concerted efforts to address the structural factors that cause inequality which lie outside of health systems’ remit (theme 5), but expansion of psychological provision is needed otherwise prescribing of APM is only going to rise, given HCPs have little other option (theme 2).

The need for better integrated care of patients with multimorbidity is apparent, and collaborative working between teams can improve outcomes, but often this only works when funding mechanisms provide the impetus for different services to work together efficiently and break down organisational silos (theme 5); this has led to the development of integrated care systems in some UK countries to redistribute funding [61]. Interesting studies have been reported in Norway on benefits of collaborative care via co-location of GP and mental health teams; while co-locating services was not mentioned in our results, examination whether benefits exist from co-location of collaborative teams for patients prescribed APM is an interesting avenue for future UK research [30].

Care could be improved if communication between services became more efficient (theme 3). Issues with preventable patient harm occurring during the transition of care at hospital discharge have been recognized [62]. Difficulty interpreting events occurring during a period of hospitalization has been reported by GPs as a significant workload challenge [63]. Use of EHR to better code, manage and share information between services is shown to improve care outcomes for patients [64]. When EHR access to clinicians is limited and information is not visible, the opportunities to improve integrated care which crosses traditional ‘specialty’ divides (such as patients with co-morbid physical and mental illness) are diminished. There is an opportunity for Artificial Intelligence to better integrate information across different EHRs, while protecting patient confidentially in the absence of a common EHR, an evolving area that is a key research priority for UK health services [65]. Opportunities for direct communication between GPs and psychiatrists, via simple solutions such as a dedicated professional telephone line for the ‘duty’ psychiatrist and GP in each clinic, would ameliorate the frustration many practitioners experience attempting to discuss care between professional colleagues (theme 3). This should be a managerial priority.

### Addressing low expectations of HCP and stigma towards those prescribed APM

Lowered expectations towards patients with SMI (theme 4) is not a new finding. Scepticism that patients taking APM will take an interest in their physical health seen in our findings, and this was also reported by Jakobs et al [44] when examining how some Dutch GPs treat patients taking APM. Discrimination against people with mental illness remains endemic in many societies, and negative attitudes by some HCPs act as a barrier to care, with patients less likely to participate in decisions regarding care [66,67]. Promisingly, some HCPs are motivated to advocate for these patients. Improved training of HCP around SMI discrimination, unconscious bias and stigma has been shown to improve care [68], but research to identify factors influencing the development of stigmatising or empathic attitudes in HCPs towards patients taking APM is needed.

### Fear of professional censure is driving defensive practice in clinicians managing patients prescribed antipsychotics

Fear of professional censure in HCPs, acting as a barrier to deprescribing of APMs, was a new finding that emerged during analysis (subtheme 5c). The wider medicolegal and regulatory milieu in which HCPs work is leading to defensive practice. Patients remain on APM due to HCP fear of censure if they stop them and a catastrophic event subsequently occurs (e.g., suicide or homicide committed by their patient). A recent study of Italian psychiatrists confirm 60% practice defensive medicine (making clinical decisions to lower risk of medicolegal liability, rather than for the benefit of the patient) and causes stress in practitioners [69,70]. Psychiatrists and GPs in the UK perceive they face ‘multiple jeopardy’ if their patient with mental illness dies by suicide or commits homicide, with censure possible from several bodies: (1) their employer; (2) the coroner; (3) civil malpractice claims; (4) criminal conviction; (5) the medical regulator (General Medical Council (GMC)). Specific advice from the GMC regarding assessment of APM risk/benefits to empower deprescribing does not exist in their prescribing guidelines [71], and GMC guidelines for ‘good medical practice’ offer no specific guidance to clinicians how to reduce risk of censure should a rare catastrophic event occur [72]. Regulators must reassure HCPs that their actions are proportionate. Some UK clinicians have lost confidence in a fair process; recent issues such as criminal convictions for gross negligence manslaughter and regulatory action against health professionals working in pressured health systems, both subsequently overturned at judicial appeal, have damaged the credibility of the GMC and judiciary [73]; a criminal prosecution of mental health professionals is currently underway. A debate on the consequences of ‘multiple jeopardy’ in the medicolegal and regulatory system, causing defensive medicine that may inadvertently harm more patients than it protects, is required.

### Conclusions

There is considerable risk that the increasing use of APM in society is inadequately managed. Unless health services address factors that contribute to fragmented and suboptimal care for patients taking APM, increasing rates of cardiometabolic disease will be difficult to ameliorate. Fear of professional censure in HCPs is a new factor emerging as a barrier to deprescribing of APM, and this needs further examination in research studies. Many practical changes are possible locally to improve integrated care for patients taking APM by health service organisations. However, other issues such as inadequate availability of non-pharmacological alternatives to APM, socioeconomic factors increasing mental distress and HCP competency gaps require national leadership by key stakeholders and politicians to improve patient care.

## Supporting information

S1 File Semi-structured interview guide. (DOCX)

S2 File Supporting quotes. (DOCX)
