# Supplementary material for: Management of antipsychotics in primary care: Insights from healthcare professionals and policy makers in the United Kingdom
Source: PLoS One. 2024 Mar 1;19(3):e0294974. doi: 10.1371/journal.pone.0294974 (PMC10906843; doi:10.1371/journal.pone.0294974)
Supplement: S1 File — (DOCX) [file pone.0294974.s001.docx]

**Supporting Information File 1:**

***Semi-structured interview guide***

| **Topic Guide Area** | **Health care professionals** |
| --- | --- |
| **Introduction** | Introduction, explain about confidentiality, right to withdraw or stop at any time. Establish background, role and if any experience in GP and psychiatry. |
| **Warm up questions** | Tell me about mental health care in your area and your role in providing support to people who are prescribed APM |
| **Why taking part?** | Can you tell me about the experiences that motivated you to participate in this study? |
| **Access issues** | How easy is it to manage APM for your patients? Do you have easy access to all the information (e.g., history, investigations) to manage the patient? Can you access support easily if concerned?? |
| **Care experience** | Have you had any postgraduate experience of working in opposite speciality? (GP or psych).  What is your experience of managing APM in your care setting? Did you have specific training in APM (for GPs and psychiatrists) that equipped you with these skills? Do you feel GPs have the skills and training? Do you feel you have the skills/resources to do this appropriately on your own? Do you feel psychiatrists think you have the skills? |
| **Experience of taking antipsychotics and managing any adverse effects of APM** | Have any of your patients had adverse effects from taking APM (eg symptoms, new onset diabetes, CVD, obesity). How did you address these? |
| **APM optimisation or trial reduction experiences** | Have you ever had to switch an APM? How did you do this? Would you be confident doing a switch of APM alone, or of a trial reduction? If not, what would you need? Have you felt you would like to, but cannot do this? Why? |
| **Thoughts/concerns about long-term APM** | What are your thoughts on patients who take APM long-term? Do you feel this is beneficial? Are you hopeful of being able to optimise, reduce or stop them? Are you concerned about any effects of APM long term? How do you balance the physical and MH risks? Which do you feel take precedence if any? |
| **Being discharged to primary care only for APM management** | How do you feel about psychiatry discharging patients to primary care to manage APM long term? Are there any benefits or drawbacks? What would improve the care for patients? |
| **Wider needs of patients and professionals** | What do you feel would improve the care for patients who need to take APM long term in terms of services, your training needs, or resources? |
| **Summary/closing** | Are there any areas you feel we haven’t covered that would help us understand better how to help patients and staff better provide care? |
| **Debrief** | Offer of resources and contact numbers |

| **Topic Guide Area** | **Managers and Directors of Policy** |
| --- | --- |
| **Introduction** | Introduction, explain about confidentiality, right to withdraw or stop at any time. Establish background, role and if any experience in GP and psychiatry. |
| **Warm up questions** | Explain their role either as a service director, or policy maker.  What is your initial impression of the ‘setting the scene’ statement? |
| **Why taking part?** | What motivated you to take part in the study? |
| **How are services doing now?** | What are your thoughts on how well services are meeting the needs of patients who need to take long term antipsychotics? |
| **How does organisation & policy contribute to the situation?** | What are your views on how organisational structures (eg NHS organisational structure, primary and secondary care interface) and on mental health policy development have helped or hindered care? |
| **What are the challenges for your organisation to improve care for mental health patients and those taking long-term APM?** | What do you find works well, what limits your capacity to make change in your organisation? How well can you influence policy? What resources do you need? |
| **Policy making effects (eg Mental Health Measures)** | Can you think of how mental health policy (e.g. Mental Health Measures) has improved or impaired care for this group of patients? |
| **Who makes policy?** | Are you aware of who is making or influencing mental health policy? How are you involved (if at all). How are participants selected? Who is making the decisions? Who is not at the policy-making table who needs to be? |
| **What do you think needs to happen to change services for the better for these patients?** | If you could make 3 quick wins to change how services in your organisation (or influence policy) to improve care, what would they be? |
| **What are you going to do differently?** | Are you planning on making any changes to your working/organisation following this interview? |
| **Summary/closing** | Any thoughts or areas that you think are important that have not been covered that may impact on service delivery or policymaking? |
| **Debrief** | Offer of resources and contact numbers |
