# Supplementary material for: Management of antipsychotics in primary care: Insights from healthcare professionals and policy makers in the United Kingdom
Source: PLoS One. 2024 Mar 1;19(3):e0294974. doi: 10.1371/journal.pone.0294974 (PMC10906843; doi:10.1371/journal.pone.0294974)
Supplement: S2 File — (DOCX) [file pone.0294974.s002.docx]

**Supporting Information File 2:**

**Supporting quotes.**

| **Theme 1: Confidence of HCPs around holistic management of APM** | |
| --- | --- |
| **Subtheme** | **Supporting Quotes** |
| GPs reluctant to manage APM without psychiatrist support | *“I'm quite confident about antidepressants for switching and swapping, but not with antipsychotics… I probably would involve secondary care if a patient needed to change.”* (GP-02).  *“Hand on heart, I have probably started risperidone in a couple of patients with emotional dysregulation, just because there has been no other option, as the waiting list has been 9 or 10 months.”* (GP-04)  *“They're not stable, are they, if you're looking at changing [the APM] ...I would even be uncomfortable really with advice because I wouldn't feel as though they'd had the full assessment from the secondary care team.”* (GP-09)  *“[GPs] do not actively manage antipsychotics... They treat them as a secondary care medication... and they don't really adjust... I think that's true for patients that are discharged from secondary care.”* (PSY-07)  *“They get called in for the medication review and we try and do the blood tests...thinking about stopping or trialling off antipsychotics... is probably not something that we do.”* (MDP-05) |
| Psychiatrists reluctant to address cardiometabolic risk | *“Most people would be reluctant [to address physical problems] without advice.”* (PSY-02)  *“They seem to do the body mass index and the blood pressure. But if they're abnormal, they don't seem to do anything about it.”* (MDP-11)  *“It's quite risky. I've been bitten... changing medications for patients because of physical problems...[patients] ended up getting hospital admission, who had been stable.”* (PSY-05)  *“In psychiatry terms, it's easier to think of the risk of the short-term effect... than worry about somebody having a heart attack in a decade.”* (GP-07)  *“I think the GP can recommend or prescribe exercise as well...I've never done it...when I came here, I was [told] 'you ask the GP to do that’.”* (PSY-08)  *“With every psychiatrist I would have... a life coach, who could actually look at people holistically and help them address the other areas. The non-medical side of it. The second thing I would do is have a GP in that clinic.”* (MDP-04) |
| Gaps for HCP in postgraduate and post-speciality training | “N*ot everyone on a GPVTS does psychiatry. There were some lectures...but I think it was limited. People on the GPVTS...weren't really trained in psychiatry.”* (MDP-11)  *“I've supervised [respondent is a GP trainer] doctors who've come through psychiatry, and they haven't found that post particularly useful. They don't get involved in that sort of decision making.”* (MDP-10)  *“I don't think there was much training in these sorts of things in postgraduate psychiatry... I certainly found that keeping up to date with things was just impractical*.” (PSY-02)  *“Whether you could have, I don't know, a shared training program where there's overlap between psychiatry and general practice, it would seem to be a nice one to have a degree of overlap with.”* (GP-03).  *“Very much my interaction with psychiatry and education is they've been teaching us, rather than a joint holistic approach to managing patients between primary and secondary care*.” (GP-02) |

| **Theme 2: Service pressures ‘trapping’ patients on antipsychotics in primary care** | |
| --- | --- |
| Limited psychology provision | *“Quetiapine [prescribing] is increasing because we are using it off-licence a lot...faced with an agitated, depressed, and aggressive patient... in the absence of psychological interventions to address those issues, you fall back on medications.”* (PSY-05) |
| Pressure to discharge patients to primary care | *“The lack of continuity... is harming people's care, because there's a move to discharge people...there is pressure ‘not to review people’ because there isn't the resource...for budgetary rather than clinical reasons.”* (GP-01)  *“Increasing requests for patients to be discharged… those ones who are just in primary care - are unlikely to ever really get off those medications... I'm not accepting those patients into primary care with a view to stopping those antipsychotics... that's an indefinite prescription.”* (GP-09)  *“We shouldn't be discharging people on drugs like olanzapine to start with...we should be holding them on the secondary care caseload, if nothing else, just for an annual review and an annual check in with those patients, and so the physical monitoring as part of the review.*” (MDP06) |
| Difficulty to get psychiatric review for patients with cardiometabolic risks | *“Apart from the frankly psychotic [patients] who could possibly need admission, we hardly get any involvement with secondary care...  It's very difficult to access and quite frustrating.”* (GP-06)  *“[Psychiatrists] response would be to just say, ‘exclude all physical causes’ - even if you've answered that question.”* (MDP-11)  *“I think that there is a lot of scope in medicine for people who sit between two specialties. You know, you could have GPs with a special interest in mental health. I think that kind of role would be something I'd like - I kind of do it now, but in terms of with more back up and working in the inpatient area. You know, in the same way that you could have acute breathlessness specialists with cardiorespiratory illness. I think much of medicine these days has become so specialized, and so pathway driven, that we forget the bits between the paths, and I think that is where the danger lurks.”* (GP-04) |

| **Theme 3: Communication between primary and secondary care** | |
| --- | --- |
| Inadequate information in written handover | *“I've just seen one patient [who] was discharged from the psychiatric services on [APM], and there is no plan.”* (GP-11)  *“I can't remember specifically being told [psychiatrist initiated an APM] was prescribed off-licence.”* (GP-05)  *“Not on weaning, but the majority have something like, ‘you can increase the quetiapine to say, 300mg... over a period of time.’ “(*GP-04)  *“I can't recall receiving information about reducing the dose... And that's the concern amongst GPs... patients are going to end up on [APM] long term... for life in certain cases.”* (GP-11)  *“The communication that we get is very patchy and often a list of commands...they are not great at understanding the governance around a shift of responsibility.” (MDP10)*  *“One case that I saw very recently, which was an elderly gentleman.... his psychiatry team said he's stable enough to reduce his [APM] and discharge... but didn't then give any sort of plan to reduce it*.” (GP-07)  *“General practices have an IT system...set up to provide a summary...the quality provided is variable.”* (PSY-07) |
| Difficulties in verbal communication between primary and secondary care | *“I honestly can't remember the last time I spoke to a psychiatrist [Laughs]. Look, I'm not being flippant. I haven’t spoken to a psychiatrist for four years.”* (GP-01)  *“Lots of advice about drugs that I don't feel are suitable or we are unable to start in primary care.”* (GP-04)  *“I think anything that develops that kind of one-to-one relationship and kind of creates, though again, I'm trying to hark about old times being great. But one thing we miss from years gone by, is that kind of doctor-to-doctor knowledge, you know, you have the mobile number of a colleague that you can call and say, “What do I do about this very quickly?” Rather than having to do a lengthy advice and guidance or referral form. And you know, we've all kind of been trapped in the way things are developed, and we say anything that fosters relationships can only improve working together.” (MDP-09)* |
| Poor integration of IT systems used in health services | *“There's the patchy I.T. infrastructure ... a bit of a hodgepodge.... If we were able to connect reliably, that would be good.” (PSY-03)*  *“If we do the blood tests here, it won't necessarily go onto the GP system...we have some wait for the paper results, or my secretary will have to call [the laboratory]. "* (PSY-08)  *“We need to get that combined input and then write-back access direct into general practice notes.”* (MDP04)  *“We've been involved in discussions with psychiatry and imminently psychiatry services within [a health region] are going to have [a common GP EHR] system. And that will enable [psychiatrists] to start initiating some of the prescriptions, whereas previously they have tried to get us to do, the cross-titrations, and things like that, which has felt very risky. And the communication hasn't been great: like I say, it's not just about what's to start, it's about what's to stop as well, and that's not made very clear.”* (MDP10)  *“I think we have got these boundaries drawn and we need to perforate the boundaries to allow that. The other thing really that would facilitate is a common clinical system that can share coded data to say that an intervention has happened, because not only have we got patients who don't get the physical health care assessments that they require, we've also got patients who will be approached by both parties and yeah, and duplication of blood pressure.”* (MDP09) |

| **Theme 4: HCPs have low expectations of patients taking APM** | |
| --- | --- |
|  | *“Do patients understand the importance of monitoring and why we need to do it?... I think we've got to do an awful lot more to empower our patients.... I do get really concerned that we discriminate against individuals with mental illness... I'm not saying every GP practice does it, but I do see it.”* (MDP-08)  *“Lots of people struggle with motivation, either they won't go to the appointment, or some of them might find it very hard to follow it up, if the medication is making them feel hungry... they often don't have the skills to cook healthy food. It makes things much more difficult.”* (PSY-06)  *“These patients don't even know the danger of the drugs that they've been given... they're not prescribed them with informed consent at the beginning. I mean, clearly, if you're psychotic and under the care of the Mental Health Act, that's not relevant. But you need to know the consequences of these drugs.”* (GP-08)  *“If I'm really honest with you... I don't think they are [informed about risks].”* (PSY-04)  *“It's a challenge to get the patients to engage... it's not uncommon for patients to refuse investigations... it becomes quite difficult to have done the stuff that you know should be in place before prescribing.”* (PSY-01)  *"[It’s] a little bit challenging to get them to engage...that is probably very neglectful of their health…. we're doing a lot of chasing, and a lot of them are not that bothered about coming in....a lot of them are quite ‘doctor avoiding’.”* (GP-02)  *“Say I've spotted [cardiometabolic concerns] in the patient, so I will ask the GP to follow that up. Well, we know they’re not going to go and see their GP!”* (PSY-01) |

| **Theme 5: Strategic factors affecting APM prescribing in primary care** | |
| --- | --- |
| Contractual divisions between primary and secondary care | *“There's clearly a problem...part of it is about organizational boundaries...but also it is this very, very definite divide between physical and mental health, which is more of a cultural challenge.”* (MDP-07)  *“Maybe a third of [patients taking APM] in my practice, I reckon are not on any QOF register?”* (GP-08)  *“The conditions to be on that [QOF mental health register] is quite narrow...so that would not actually capture a lot of those patients.”* (GP-11)  *“Schizophrenic patients have almost dropped off our view, especially where people are quieter as opposed to the ‘noisier’ patient groups.... they get the political attention, even if their clinical presentation doesn't warrant that level of intervention.”* (MDP-06)  *“Even though we are all one footprint, we still operate in silos, a lot of the time because of budgets. We need to do that for budgeting purposes...I want to make them a bit leakier... and use our full resources for the benefit of our patients. Setting targets for the cluster, to make sure that every patient with SMI has had a physical health check this year.... The difference is that almost everything, apart from quality improvement aspects, has been absorbed within the core GMS contract. So, all the data gathering around chronic disease, long-term and condition management, including SMI, on the assumption now is that it's done within the core contract, so how we monitor that is incredibly challenging?”* (MDP-09)  *“How do we create multi-professional teams to be able to support people or recognize that this may be an outcome. So, who else do we need to draw in to support patients? And I think this is probably more of a conceptual issue that goes to the heart of our health care system: Are we about fitting people into the existing system or fitting to see the system around people?” (MDP02)* |
| Worsening inequality driving antipsychotic use | *I think we're using [APMs] to 'hold' people who we really shouldn't be on them.”* (MDP-06)  *“There was a really good project, especially with care home patients, to ensure that the care home staff were educated in these matters. And it was quite heartening to visit care homes and learn that they were telling us, 'Well do you think you shouldn't be doing this kind of things? Don't you think that this medication shouldn't be prescribed for this patient?'”* (GP-11) |
| Medicolegal and regulator fears by HCPs. | *“The answer would be no way [to stopping] …from a legal standpoint, [we are] not operating in an area that is our expertise - if things were to go wrong with a patient harming someone, you'd feel a bit at risk.”* (GP-01)  *“I'm…reluctant to change [antipsychotics]: the consequences of relapse can be really bad.”* (PSY-05)  *“I've never known a practitioner to be brought before a regulatory body for trying to do …the very best for a patient? But yes, it might be the case the regulator is felt too harsh, it really might be.”* (MDP-06)  *“We're just employing an advanced nurse practitioner on the adult mental health register. But you know, we want that individual to work across the transition age...and the real issue we are hitting is that, because they're registered on the second part for adults, and not the children's part, they can't work across that transition age spectrum. So, I think that's an example where an individual has the skill set, the training is there, but the register and regulatory system gets in the way.”* (MDP-06)  *“Clearly, we don't want rogue people prescribing anything they like off a formulary because they've read something in the paper about it. But equally, we don't want to make it so inhibitory that people are frightened and say, "I dare not start a very, very safe blood pressure tablet because it's not something that my other colleagues do, so I'm not doing it". What happens is that patient never gets the blood pressure treatment and then they have a stroke early.” (MDP-06)* |
